# Iron-Responsive Olfactory Uptake of Manganese Improves Motor Function Deficits Associated with Iron Deficiency

**DOI:** 10.1371/journal.pone.0033533

**Published:** 2012-03-30

**Authors:** Jonghan Kim, Yuan Li, Peter D. Buckett, Mark Böhlke, Khristy J. Thompson, Masaya Takahashi, Timothy J. Maher, Marianne Wessling-Resnick

**Affiliations:** 1 Department of Genetics and Complex Diseases, Harvard School of Public Health, Boston, Massachusetts, United States of America; 2 Department of Pharmaceutical Sciences, Massachusetts College of Pharmacy & Health Sciences, Boston, Massachusetts, United States of America; 3 Department of Radiology, Beth Israel Deaconess Medical Center, Harvard Institutes of Medicine, Boston, Massachusetts, United States of America; INSERM/CNRS, France

## Abstract

Iron-responsive manganese uptake is increased in iron-deficient rats, suggesting that toxicity related to manganese exposure could be modified by iron status. To explore possible interactions, the distribution of intranasally-instilled manganese in control and iron-deficient rat brain was characterized by quantitative image analysis using T1-weighted magnetic resonance imaging (MRI). Manganese accumulation in the brain of iron-deficient rats was doubled after intranasal administration of MnCl_2_ for 1- or 3-week. Enhanced manganese level was observed in specific brain regions of iron-deficient rats, including the striatum, hippocampus, and prefrontal cortex. Iron-deficient rats spent reduced time on a standard accelerating rotarod bar before falling and with lower peak speed compared to controls; unexpectedly, these measures of motor function significantly improved in iron-deficient rats intranasally-instilled with MnCl_2_. Although tissue dopamine concentrations were similar in the striatum, dopamine transporter (DAT) and dopamine receptor D_1_ (D1R) levels were reduced and dopamine receptor D_2_ (D2R) levels were increased in manganese-instilled rats, suggesting that manganese-induced changes in post-synaptic dopaminergic signaling contribute to the compensatory effect. Enhanced olfactory manganese uptake during iron deficiency appears to be a programmed “rescue response” with beneficial influence on motor impairment due to low iron status.

## Introduction

Divalent metal transporter-1 (DMT1) mediates uptake of manganese across the olfactory epithelium into the brain [1]. It is also the major transporter for iron absorption in the duodenum [2], [3]. During iron deficiency, the transporter's expression becomes up-regulated in both olfactory and intestinal epithelia [1], [2], [4]. Thus, up-regulation of DMT1 in iron-deficient rats is associated with increased olfactory manganese uptake [1]. The physiological significance of iron-responsive manganese transport to the brain has not been explored. Enhanced manganese delivery to the brain promoted by iron deficiency could have a toxic impact by modifying neurological complications of poor iron status. Iron-deficient animals are hypoactive [5], [6], and decreased physical activity and impaired skeletal motor activity are thought to be due to altered dopaminergic function [5], [7]–[11]. Manganese toxicity is also known to cause motor deficits, and locura manganica or “manganese madness” is associated with bradykinesia, rigidity, tremor and dystonia [12].

We speculated that impaired motor activity due to iron deficiency might be negatively influenced by olfactory manganese exposure due to iron-responsive uptake of the metal across the air-brain-barrier. We therefore determined the distribution of intranasally-instilled manganese in the brain of control and iron deficient rats using magnetic resonance imaging (MRI) and examined the functional interactions between manganese exposure and iron deficiency, both of which can impair motor function. Unexpectedly, the impaired motor function of iron-deficient rats was corrected by olfactory manganese instillation. These effects were associated with manganese-induced changes in dopamine receptors and transporters that suggest altered post-synaptic signaling compensates for motor impairments due to iron deficiency. Iron-responsive manganese assimilation in the brain serves as a “rescue response”.

## Results

### Manganese instillation of iron-deficient rats

Iron deficiency was induced in weanling Sprague-Dawley rats fed an iron-deficient diet (5 mg iron/kg) for 4 weeks. Physiological and hematological parameters were evaluated at 7 weeks as shown in **Table 1**. Compared to age-matched rats fed control chow (220 mg iron/kg), rats fed the iron-deficient diet weighed 11% less (220 *vs* 248 g; *P*<0.05) although brain weight was similar between both groups. Hematocrit values were significantly reduced (23.9 *vs* 44.1%; *P*<0.05) and non-heme iron levels in liver and serum were lower (8.2 *vs* 43.2 µg/g and 0.28 *vs* 1.34 µg/mL, respectively; *P*<0.05).

**Table 1:** Physiological and hematological characteristics of rats treated with olfactory manganese under iron deficiency.

	Control												Iron-deficient											
MnCl_2_ (mg/kg)	0			(N)	30			(N)	60			(N)	0			(N)	30			(N)	60			(N)
Body weight ‡, g	248	±	6	(9)	238	±	8	(9)	239	±	4	(4)	220	±	6	(10)	198	±	7	(9)	201	±	3	(4)
Brain weight, g	1.87	±	0.08	(5)	1.82	±	0.06	(5)	1.94	±	0.03	(4)	1.80	±	0.03	(7)	1.81	±	0.04	(5)	1.82	±	0.01	(4)
Hematocrit ‡ ^,^ §, %	44.1	±	1.1	(9)	40.8	±	0.8	(9)	45.8	±	1.6	(4)	23.9	±	1.3	(10)	24.1	±	0.6	(9)	20.9	±	0.9	(4)
Liver non-heme iron ‡, µg/g liver	43.2	±	2.2	(4)	44.5	±	6.4	(3)	44.0	±	4.6	(4)	8.2	±	0.3	(4)	8.3	±	1.4	(3)	7.2	±	0.9	(4)
Serum iron ‡, µg/mL	1.34	±	0.16	(4)	ND		1.37	±	0.12	(4)	0.28	±	0.07	(4)	ND		0.36	±	0.11	(4)				

Data are presented as the mean ± SEM and were analyzed by two-way ANOVA; ND, not determined;

‡ *P*<0.05, effect of iron deficiency;

§ *P*<0.05, effect of interaction between MnCl_2_ and iron deficiency.

Two manganese dosing regimens were studied with the maximum soluble amount (10 mg MnCl_2_/kg) instilled either 3 times across one week or 6 times across 3 weeks (total doses of 30 mg/kg or 60 mg/kg). Separately matched control and iron-deficient diet cohorts were intranasally-instilled with vehicle alone (distilled water). Intranasal instillation did not change body or brain weight (**Table 1**). Hematological parameters were also unaffected, although a slightly reduced hematocrit value was noted in iron-deficient rats administered 60 mg MnCl_2_/kg compared to water-instilled iron-deficient rats (**Table 1**).

### Effect of iron deficiency on brain accumulation of intranasally-instilled manganese

**Figure 1:**
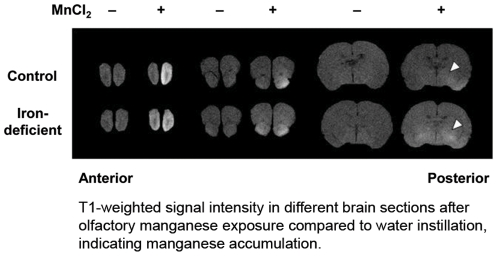
Manganese accumulation in the brain after intranasal instillation. MnCl_2_ was instilled into the right nostril of rats three times on days 1, 4, and 6 (30 mg/kg). Distilled water was used as a vehicle control. Two days following the last instillation, MR images were taken from isoflurane-anesthetized rats using a 4.7T MR device to assess signal intensity on T1-weighted images of axial sections of the brain with a thickness of 1 mm. Arrowheads indicate the striatum.

Magnetic resonance imaging was performed on isoflurane-anesthetized rats using a 4.7T MR device to assess signal for brain manganese on T1-weighted images. **Fig. 1** presents the T1-weighted images in axial sections for control and iron-deficient rats intranasally-instilled with water or 30 mg MnCl_2_/kg two days following the last instillation. Greater signal intensity in the brain, particularly the olfactory bulb and the basal ganglia, was observed in manganese-instilled rats compared with water-instilled controls, confirming the signal is associated with the metal's accumulation. Cross-over of manganese from the right nostril (instillation site) to the left hemisphere was more pronounced in iron-deficient rats, with greater accumulation appearing in posterior regions including the striatum (**Fig. 1** **, arrowheads**).

**Figure 2:**
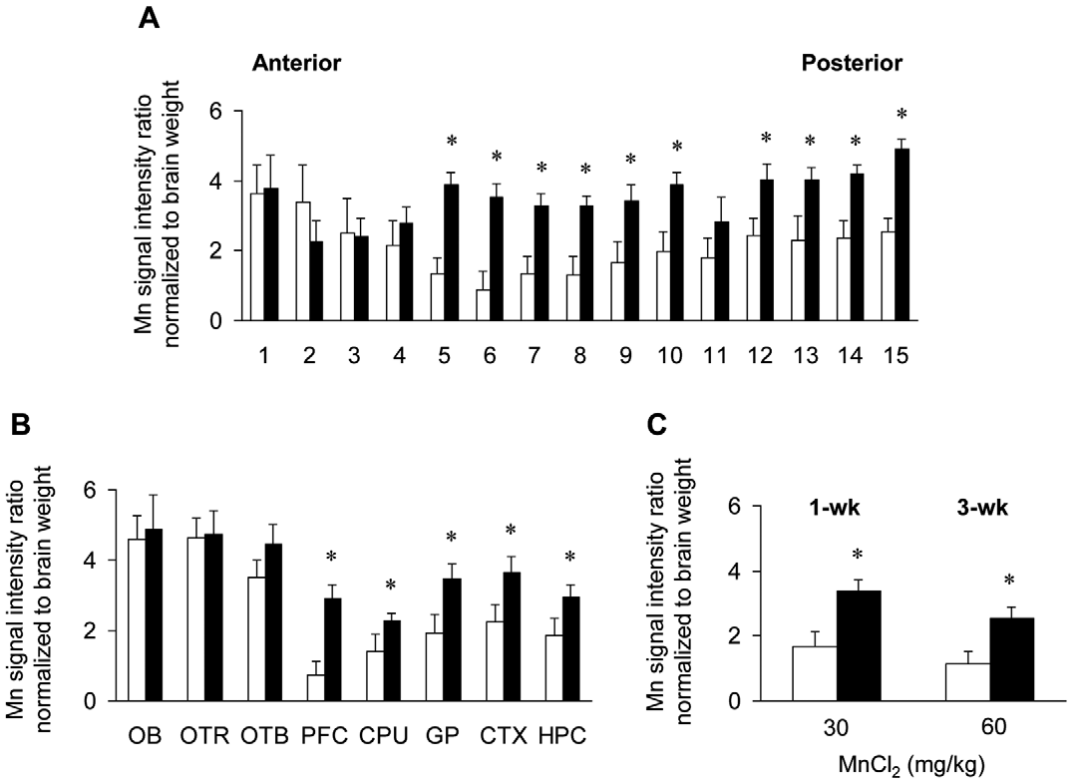
Effects of iron deficiency on manganese accumulation in the brain after intranasal instillation. After intranasal instillation of MnCl_2_ (3×10 mg/kg for 1 wk or 6×10 mg/kg for 3 wks), a signal intensity ratio of brain to the background for each image was calculated and corrected for endogenous signal intensity of respective diet group and then normalized to brain weight and dose. Manganese distribution in the axial sections of the brain tissue (**A**), in specific brain regions (**B**) and in the whole brain integrating all sections (**C**) was compared between control and iron-deficient rats. Empty and closed bars represent water-instilled and MnCl_2_-instilled rats, respectively. Data were presented as mean ± SEM (N = 4–5). * *P*<0.05 between control and iron-deficient rats determined by two-sample *t*-test. OB, olfactory bulb; OTR, olfactory tract; OTB, olfactory tubercle; PFC, prefrontal cortex; CPU, caudate-putamen or striatum; GP, globus pallidus; CTX, cortex; HPC, hippocampus.

To directly compare the distribution of manganese in the brain between control and iron-deficient rats, the signal intensity on the T1-weighted images was corrected for the endogenous signal intensity of the respective water-instilled diet group and normalized to brain weight (**Fig. 2**). Signal intensity due to manganese in the axial sections of the brain tissue was significantly enhanced in an anterior-to-posterior fashion (**Fig. 2A**). Iron-deficient rats displayed increased signal in specific brain regions including prefrontal cortex, globus pallidus, and hippocampus. Although regions closer to the nasal instillation site (i.e., olfactory bulb and tract) showed a similar trend, the difference in signal was not statistically significant (**Fig. 2B**). The combined signal intensity calculated after integrating signal across all MRI sections (15×1 mm) of the whole brain was doubled in iron-deficient rats instilled with either 30 or 60 mg MnCl_2_/kg (**Fig. 2C**, 3.36 *vs* 1.67 or 2.55 *vs* 1.16 intensity ratios, respectively; *P*<0.05). The fact that signal intensity did not increase at the higher dose suggests a saturation effect on olfactory uptake and/or accumulation of metal. It should be noted that administered doses were based on body weight, and intensity ratios may actually under-estimate the true difference between control and iron-deficient brain manganese accumulation, since brain weights were similar between the two groups. The fact that manganese is eliminated very slowly from the rat brain with a half-life >50 days [13], [14], suggests that elevated levels of manganese in iron-deficient rats result from enhanced olfactory uptake rather than from reduced clearance from the brain. However, other possible explanations of these data include the fact that the MRI signal intensity may become saturated such that higher Mn concentrations cannot be distinguished. Alternatively, neurotoxic effects of Mn on the transport process itself may interfere.

### Effect of iron deficiency and olfactory manganese instillation on motor coordination

**Figure 3:**
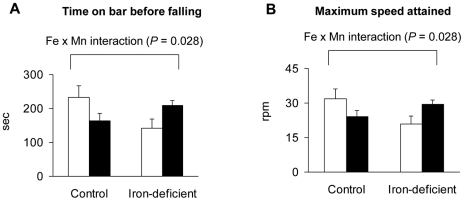
Effect of iron deficiency and manganese exposure on motor coordination of the rat. Rats were pair-fed, intranasally instilled (6×10 mg/kg) for 3 wks, and tested on the rotarod device to record the time to falling-off (**A**) and speed of the rod (**B**). Empty and closed bars represent water-instilled and MnCl_2_-instilled rats, respectively. Data were presented as mean ± SEM (N = 3–4 per group) and were analyzed using two-way ANOVA.

To examine functional interactions between iron deficiency and manganese exposure, a motor coordination study was carried out using a standard rotarod device. Control and iron-deficient rats were instilled with 60 mg MnCl_2_/kg or vehicle over 3 weeks as described above, then tested for motor function and Fe x Mn interactions. Manganese-instilled rats fed the control diet displayed diminished motor coordination, however, iron-deficient rats instilled with manganese stayed on the bar longer before falling and displayed an increase in the maximum speed attained on the rotarod (**Fig. 3** **, A and B**). The effect of Fe *x* Mn interaction was significant (*P* = 0.028) and showed an unexpected reversal of motor impairment.

### Effect of olfactory manganese exposure and iron deficiency on striatal dopamine levels

**Figure 4:**
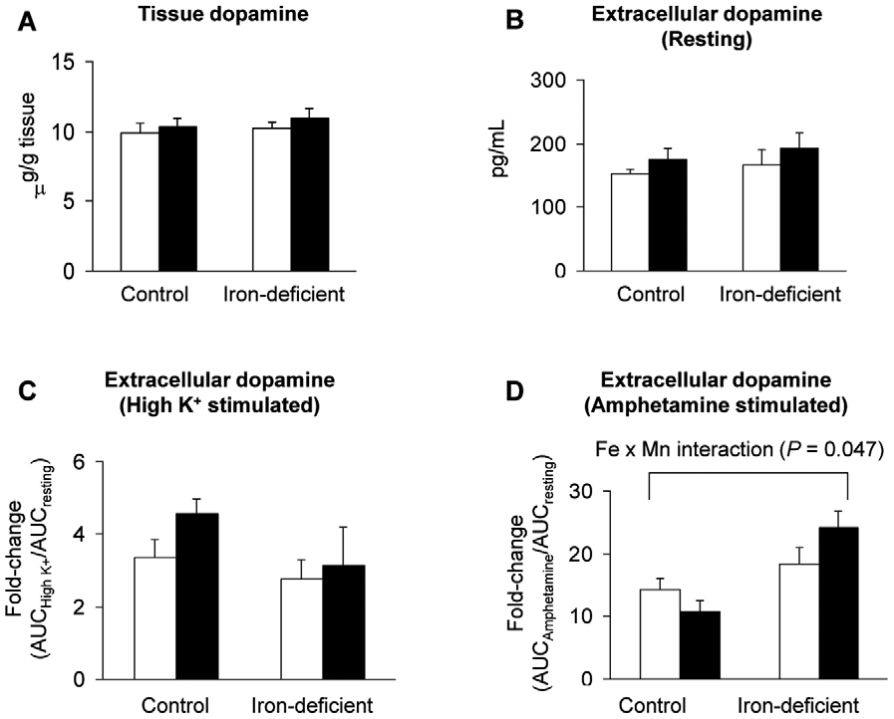
Effect of iron deficiency and manganese instillation on dopamine turnover in the striatum. Immediately after rotarod test (**Fig. 3**), rats were euthanized and striatal tissues were homogenized to determine the tissue concentrations of dopamine (**A**; N = 3–4). Rats of another cohort were anesthetized with urethane and microdialysis was performed to determine the basal dopamine concentration at resting state (**B**; N = 6–8) and the fold-change in AUC of dopamine release either after high K^+^ (30 mM) stimulation (**C**; N = 6–8) or after amphetamine stimulation (1 mg/kg, s.c.) in striatal extracellular fluid (**D**; N = 6–8). Empty and closed bars represent water-instilled and MnCl_2_-instilled rats, respectively. Data were presented as mean ± SEM and were analyzed using two-way ANOVA.

To better understand the mechanistic basis for the observed interactions between manganese exposure and iron deficiency on motor function, levels of the neurotransmitter dopamine were determined in the striatum, a brain region associated with motor control and coordination. Tissue dopamine concentrations in control and iron-deficient rats were similar and unaffected by intranasal instillation of 60 mg MnCl_2_/kg (**Fig. 4A**). Microdialysis experiments determined resting extracellular dopamine concentrations did not differ significantly among water-instilled or manganese-instilled control and iron-deficient cohorts (**Fig. 4B**). While the fold-change in K^+^-stimulated dopamine release did not differ significantly (**Fig. 4C**), amphetamine-evoked release increased extracellular dopamine levels 14-fold in control and 18-fold in iron-deficient rats. Iron-deficient rats instilled with 60 mg MnCl_2_/kg had a 24-fold increase in amphetamine-stimulated extracellular dopamine (**Fig. 4D**) with a significant Fe *x* Mn effect (*P* = 0.047).

### Effect of olfactory manganese exposure and iron deficiency on striatal dopamine transporter and receptor levels

**Figure 5:**
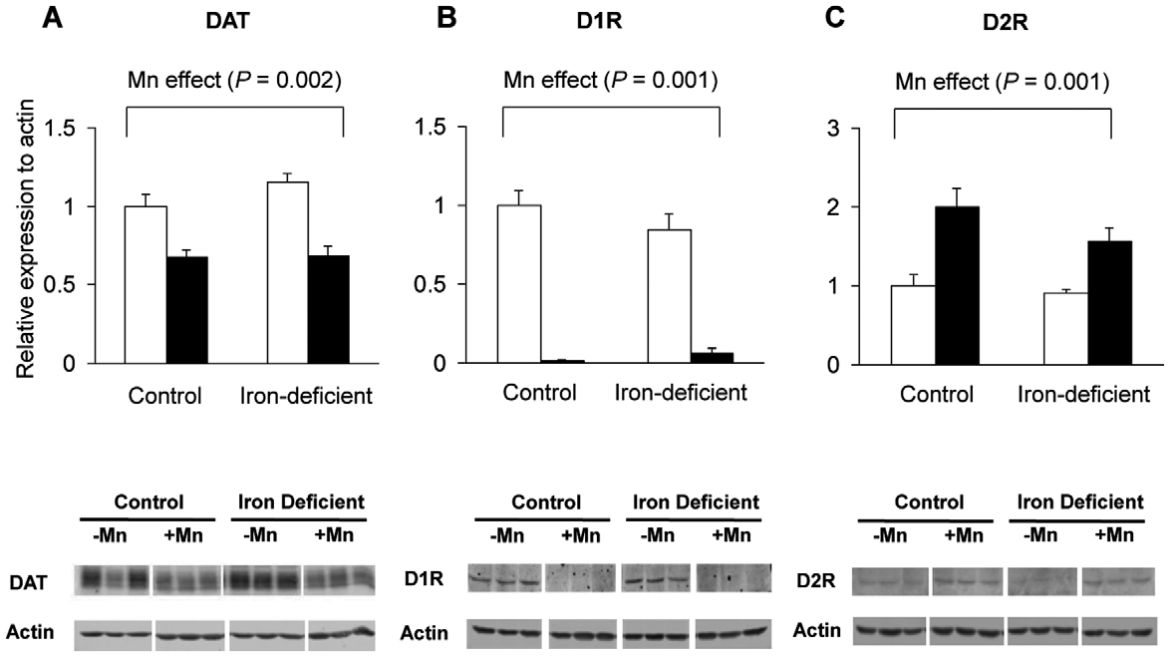
Effect of iron deficiency and manganese instillation on the expression of dopamine transporters and receptors in the striatum. Rats intranasally instilled with MnCl_2_ (6×10 mg/kg) were euthanized and striatal tissues were collected and homogenized to determine the expression levels of dopamine transporter (DAT; **A**), dopamine receptor D_1_ (D1R; **B**), and dopamine receptor D_2_ (D2R; **C**). Relative intensities of protein bands normalized to actin were determined using Odyssey software (version 2.1). Empty and closed bars represent water-instilled and MnCl_2_-instilled rats, respectively. Data were presented as mean ± SEM (N = 3–4 per group) and were analyzed using two-way ANOVA.

To determine whether manganese exposure influenced the expression of dopamine transporters and receptors in the striatum, Western blot analysis was used to quantify protein levels. Intranasal manganese instillation reduced dopamine transporter (DAT) and dopamine receptor D_1_ (D1R) levels (**Fig. 5** **, A and B**) while dopamine receptor D_2_ (D2R) levels were increased (**Fig. 5C**). These manganese-induced changes were the same in control and iron-deficient rats.

## Discussion

The most relevant route for occupational and environmental manganese exposure health effects is through inhalation [15]. Characterization of the influence of iron status on olfactory and pulmonary manganese transport has demonstrated that respiratory manganese uptake reflects iron stores [1], [16], [17] and that DMT1 is involved in absorption across the olfactory epithelium into the brain [1]. Here, we studied interactions between systemic iron status, olfactory manganese uptake, and brain manganese deposition on neurobehavioral function and dopamine neurotransmission in the striatum. Rats fed a low iron diet during the course of this study had similar brain weights, although iron deficient rats had lower body weights compared to control rats. Hematocrit, liver non-heme iron and serum iron values were all significantly lower in rats fed the iron-deficient diet. The purpose of our study was to better understand the consequences of enhanced olfactory manganese absorption under these conditions of low body iron status.

Manganese is taken up from the nasal cavity along the olfactory tract into the brain [1], [18]–[20]. Using T1-weighted MR imaging, we determined brain manganese distribution after intranasal instillation of 30 and 60 mg MnCl_2_/kg body weight. A strong anterior-to-posterior trend of manganese-associated signal intensity on T1-weighted images was observed. These results generally agree with earlier studies using radioactive tracer to map uptake and transport from the olfactory bulb into the brain [1], [18], [19], [21]. It has been proposed that divalent manganese acts as a calcium ortholog and is taken up into synaptic vesicles that undergo anterograde transport in a microtubule-dependent (colchicine-sensitive) manner for its subsequent distribution [22], which explains relatively low accumulation of manganese in the olfactory bulb in our study. Iron deficiency has been shown to increase brain manganese deposition when administered through diet or by intravenous injection thereby following a pathway across the blood-brain-barrier [23], [24]. To our knowledge, the present study is the first to use MRI to map the olfactory route across the air-brain-barrier under iron deficiency conditions, and our results demonstrate that brain manganese is doubled in iron deficient rats compared to controls after one week of intranasal instillation (total dose of 30 mg MnCl_2_/kg). Significantly greater signal intensities were observed in the prefrontal cortex, caudate putamen, globus pallidus, cortex and hippocampus. Our previous isotopic tracer kinetic study demonstrated that brain uptake of manganese after intranasal instillation was more than 20-fold greater than after intravenous injection. Combined, these separate lines of evidence indicate that manganese transport via the air-brain-barrier is by far more efficient than transfer across the blood-brain-barrier and is significantly up-regulated as a result of systemic iron deficiency [1].

Because the ratio of signal intensity to brain weight did not increase with continued dosing from 1 to 3 weeks, the mechanism responsible for metal absorption and accumulation from the olfactory pathway appears to be saturable. Increased manganese accumulation in iron-deficient rats could be explained by greater olfactory uptake of metal into the brain and/or by increased number of iron-responsive metal-binding sites. We have previously shown that olfactory uptake of ^54^Mn to the brain is enhanced by low iron status but it is significantly reduced in Belgrade rats with DMT1 deficiency [1]. It is therefore likely that manganese distribution mapped by MRI reflects changes in levels of this transporter in the olfactory epithelium, although it is also possible that manganese binding sites or other transport pathways secondary to increased manganese entry across the olfactory epithelium are enhanced by iron deficiency. Other possibilities discussed above include neurotoxic effects of manganese on retrograde transport from the nasal cavity to the brain. Further studies are warranted to examine manganese binding sites by radioactive uptake and metal distribution, for example.

Motor impairments due to iron deficiency are well documented [10] and behavioral effects due to excess dietary manganese have been reported [25], [26]. Both the time on bar before falling and the maximum speed attained on the rotarod were reduced in control rats intranasally-instilled with manganese, and both of these functions were more severely impaired in iron-deficient rats. However, the observation that intranasal manganese instillation of iron-deficient rats improved performance with a significant Fe *x* Mn interaction (two-way ANOVA, *P* = 0.028) was unexpected. This evidence points to a beneficial influence of iron-responsive manganese uptake to the brain – a “rescue response” triggered under low iron conditions.

To explore the underlying basis for behavioral Fe *x* Mn interactions, dopamine metabolism and signaling in the striatum was examined. Both high manganese [27] and iron deficiency [7], [8] alter dopamine turnover and metabolism. Under our experimental conditions, striatal dopamine levels were similar between control and iron-deficient rats. While some investigations have shown that tissue dopamine levels are altered by iron deficiency, differences in age, circadian cycle, extent of iron depletion, and duration of low iron status between various study groups have led to inconsistent results [5], [7], [10], [11], [28]. Likewise, chronic manganese exposure to the brain across the blood-brain-barrier is thought to diminish dopamine levels [29]–[31], but the olfactory exposure used in our study did not appear to perturb dopamine content, possibly because metal accumulation was lower, manganese deposition was regionally different, and/or the duration of manganese exposure (1–3 weeks) was less than previously studied [29]–[31]. We also studied changes in release and turnover of extracellular dopamine by microdialysis. It has been reported that extracellular dopamine is increased in iron-deficient animals as a consequence of reduced uptake by DAT [9], [11]. Under our study conditions, DAT levels were the same in control and iron-deficient rats, but protein levels were reduced by olfactory manganese exposure in both groups. Similar effects of manganese exposure have been reported in studies of chronic administration by diet [29] or injection [30]. Our study indicates olfactory administration of manganese can also down-regulate DAT, possibly in a more acute manner.

Consistent with reduced DAT, microdialysis measurements showed that extracellular resting dopamine was slightly increased in the manganese-exposed groups and K^+^-stimulated levels were higher, but neither of these effects was statistically significant. In contrast, extracellular dopamine increased in response to amphetamine with a significant Fe *x* Mn interaction (two-way ANOVA, *P* = 0.047). Manganese has been shown to modify amphetamine-induced responses but the precise mechanism is unknown [27]. It is interesting to speculate that the underlying basis for the observed pharmacological Fe *x* Mn interaction could reflect compensatory changes in iron-deficient rats induced by olfactory manganese exposure that restored normal activity in the motor tests performed on this group. In particular, manganese might not only modulate DAT levels but the metal could influence its function by direct competition or by other non-competitive interactions. It has been shown that some DAT inhibitors negatively affect manganese uptake by striatial tissue [32], [33], and manganese inhibits dopamine uptake by synaptosomes [32], [34].

Alternatively, olfactory manganese exposure may modulate downstream signaling by altering dopaminergic receptors. There is evidence indicating iron deficiency disrupts dopaminergic functions by reducing D1R and D2R levels [9], [28]. Although we did not observe statistically significant effects on dopamine receptor levels due to iron deficiency, olfactory manganese exposure significantly reduced D1R and increased D2R levels in both control and iron-deficient rats. These results are in good agreement with previous findings reported for gestational/lactational exposures to manganese [35], [36]. Overall, this evidence suggests that olfactory manganese exposure up-regulates post-synaptic signaling through D2R, which may compensate for loss of dopaminergic signaling under iron-deficient conditions.

Finally, it should be recognized that multiple pathways could be involved in the interaction between brain iron deficiency and the monoaminergic system, and that many metal-containing enzymes and/or cellular components contribute to this process. It is conceivable that increased availability of manganese affords the replacement of iron to rescue the loss of certain iron-dependent enzymatic or structural functions. For example, both iron and manganese have been shown to support activity of tyrosine hydroxylase, the rate-limiting enzyme for dopamine synthesis [37]. In fact, the idea that unoccupied metal binding sites are available under iron deficiency to bind excess manganese and limit the metal's toxicity is compatible with the Fe *x* Mn interactions observed in our study.

Our data confirm that both iron and manganese play active roles in modulating motor function. Contrary to our speculation that manganese might exacerbate functional losses due to iron deficiency, our study suggests iron-responsive olfactory manganese uptake confers a beneficial response to low iron status. Along similar lines, the protective nature of manganese associated with iron homeostasis and oxidative stress defense recently has emerged for bacteria [38] and yeast [39]. The pathways responsible for iron-responsive olfactory uptake of manganese and the metal's impact on dopaminergic function warrant further exploration as a possible course of therapeutic intervention for conditions wherein iron deficiency promotes functional deficits (e.g., restless legs syndrome) or loss of dopaminergic function impacts motor coordination and control (e.g., Parkinson's disease).

## Materials and Methods

### Ethics statement

This study was performed in strict accordance with the recommendations in the Guide for the Care and Use of Laboratory Animals of the National Institutes of Health. The protocol was approved by the Harvard Medical Area Animal Care and Use Committee (Animal Experimentation Protocol AEP #03769).

### Animals and diets

Weanling Sprague-Dawley rats (Taconic) were fed either control chow (220 mg iron/kg, PicoLab 5053, PharmaServ) or iron-deficient diet (5 mg/kg, TD99397, Harlan Teklad) for 4 weeks to establish control and iron-deficient cohorts [1], [16]. Both groups were fed *ad libitum* for MRI studies of brain manganese distribution. Iron-deficient rats were fed *ad libitum* for behavioral and neurotransmitter analyses while control rats were pair-fed to match body weight for these experiments. Rats were intranasally instilled with MnCl_2_ (Sigma-Aldrich) into the right nostril with thin gel loading tips under isoflurane anesthesia. Distilled water (vehicle) was used as an instillation control. Two dosing regimens (1 and 3 weeks) were studied: 6-wk-old rats were instilled with 10 mg MnCl_2_/kg three times at days 1, 4, and 6 (a total of 30 mg/kg), whereas, for three-week treatment, 4-wk-old rats were instilled with 10 mg MnCl_2_/kg twice weekly at days 1, 5, 8, 12, 15, and 19 (a total of 60 mg/kg). This dose schedule permitted sufficient distribution of manganese to the brain but because iron homeostasis is influenced by circadian rhythm, we instilled manganese in the morning (every 2–3 days). Timing was limited to short duration exposure to severe iron deficiency. At the end of the study, rats were euthanized by isoflurane overdose followed by exsanguination for collection of blood, liver, and brain tissues to analyze iron status.

### MR imaging and analysis

Anesthetized rats were placed in a 4.7T MR device and immediately imaged for 30–60 min to obtain multi-slice T1-weighted images covering entire brain in both axial and sagittal sections 1 mm thick (gapless). The echo time/repetition time used was 500/11 msec and the field of view was 3×3 cm^2^ yielding a resolution of 120 µm. Signal intensity was measured and normalized to background signal intensity using ImageJ software (NIH, version 1.44). The endogenous averaged signal intensity ratio values of vehicle-treated animals of the respective diet group were subtracted to determine signal due to instilled manganese uptake. The manganese signal intensity ratio was finally normalized to post-mortem brain weight. Manganese distribution in the brain including specific brain regions (e.g., prefrontal cortex, globus pallidus, etc), total brain tissue of each section (2-dimensional), and integration of whole brain sections (3-dimensional) was compared between control and iron-deficient rats.

### Rotarod test

Pair-fed rats (3–4 per group) instilled with water or 60 mg MnCl_2_/kg as described above were placed on a standard accelerating rotarod device (Harvard Apparatus). Following 3-day training sessions at fixed speeds, rats were tested twice on the rotarod with accelerating speeds from 4 to 40 rpm over 5-min (maximum time on bar) or until the animals fell off. Time on bar and speed attained on rotarod before falling were recorded, and the better score of the two trials was used for analysis.

### Microdialysis

Rats (6 to 8 per group) were anesthetized with urethane (1 g/kg, i.p.) and placed in a stereotaxic apparatus (Stoelting). A mid-sagittal incision was made over the skull, and a small hole was drilled to allow for implantation of a guide cannula and a precalibrated 3-mm concentric microdialysis probe (CMA-12 Elite, CMA/Microdialysis) into the right striatum (Bregma: AP+0.7 mm, LR–2.8, DV–6.8). Following probe implantation, artificial cerebrospinal fluid (K^+^ 2.7 mM) was continuously perfused (2 µL/min) with an infusion pump (Harvard Apparatus) for 2 hrs to allow for stabilization of injury-mediated release of neurotransmitters, after which 4 baseline samples (20-min each) were collected to determine resting levels of dopamine. Samples were collected for an additional 60–80 min during perfusion of fluid containing 30 mM K^+^ or after subcutaneous injection of 1 mg/kg *d*-amphetamine to stimulate dopamine efflux. Microdialysates were mixed with 5 µL of 0.1 M acetic acid containing oxalic acid (1.0 mM) and L-cysteine (3.0 mM) to prevent oxidative degradation of monoamines [40] and stored at −80° until HPLC analysis. The area under the concentration-time curve (AUC) analysis was employed to determine *d*-amphetamine-induced dopamine release [41].

### Analysis of dopamine in microdialysates

The HPLC system consisted of an ESA 542 autosampler (ESA, Thermo Fisher, Waltham, MA), an ESA 582 dual-piston pump, a Capcell PAK MG C_18_ column (50×1.5 mm, 3 µm particles, ESA), an ESA Coulochem III detector, with a 5020 guard cell and a 5041 amperometric analytical cell. The guard cell was set at a potential of +275 mV and the 5041 analytical cell was set to +220 mV. Data were acquired from the 5041 analytical cell and analyzed with EZChrom Elite software configured for the Coulochem III system by ESA. The mobile phase contained 150 mM sodium dihydrogen phosphate monohydrate and 4.76 mM citric acid monohydrate, adjusted to pH 5.6 with concentrated semiconductor grade sodium hydroxide before adding 3 mM sodium dodecyl sulfate, 50 µM EDTA, 10% methanol (v/v) and 15% acetonitrile (v/v), in NANOpure water prefiltered first through a C_18_ cartridge (Sep-Pak, Waters, Milford, MA) then through a 0.2 µm nylon membrane (Millipore, Burlington, MA). The flow rate was 0.2 mL/min and the column was kept at 29°C. Calibration was by external standards prepared in solutions of 0.2 M HClO_4_, 0.2 µM Na_2_EDTA and 0.2 µM ascorbic acid [42]. Concentration/area calibrations were linear over the concentration ranges found in the microdialysates, with *r* ^2^>0.999. The detection limit was approximately 10 pg/mL.

### Analysis of dopamine in tissue homogenates

Striatal tissue homogenates were prepared in ice cold 0.2 M perchloric acid (1∶10 w/v) containing ascorbic acid (0.2 µM) and EDTA (0.2 µM) and centrifuged for 6 min at 15,000×g. An aliquot of the supernatant was further diluted 1∶2 in perchloric acid solution and centrifuged for an additional 2 min at 15,000×g, and final supernatant (10 µL) was analyzed using an HPLC system consisting of an ESA 542 autosampler (ESA), an ESA 580 dual-piston pump, an ESA MD-150 column (C_18_, 150×3.2 mm, 3 µm particles, 120 Å pores), an ESA Coulochem II detector (Model 5200), with a 5020 guard cell and a 5011 coulometric analytical cell. The potentials of the electrochemical cells were E_GuardCell_ +350 mV, E_1_ −150 mV and E_2_ +300 mV, all against a palladium reference electrode. Data were acquired from E_2_ and analyzed by PC/Chrom+ software (H & A Scientific, Greenville, NC). The mobile phase consisted of 90 mM sodium phosphate monobasic monohydrate; 50 mM citric acid monohydrate; 1.7 mM 1-octanesulfonic acid sodium salt hydrate, HPLC grade; 50 µM disodium ethylenediamine tetraacetate dihydrate; and 10% acetonitrile (v/v) in water prefiltered as described above. The flow rate was 0.4 mL/min. Calibration was by external standards prepared in solutions of the above preservative. Concentration/area calibrations were linear over the ranges of concentrations found in the microdialysates, with *r* ^2^>0.999.

### Western blot analysis of dopamine transporters and receptors

Microdissected striatal regions were homogenized in Tris-NP40 buffer (50 mM Tris-HCl, 150 mM NaCl, 0.5% NP40, pH 7.5) containing protease inhibitors (Complete Mini, Roche). Samples (50–100 µg proteins) were electrophoresed on a 10% SDS-polyacrylamide gel and transferred to polyvinylidene difluoride membrane (Millipore). After blocking, the membrane was incubated in goat anti-dopamine transporter (DAT) antibody (1∶100, Santa Cruz), mouse anti-dopamine receptor D_2_ (D2R) antibody (1∶100, Santa Cruz) or rat anti-dopamine receptor D_1_ (D1R) antibody (1∶100, Sigma). As a control for loading, the immunoblot was also incubated with mouse anti-actin (1∶10,000, MP Biomedicals). The blots were incubated with IRDye800/680-conjugated secondary antibody (1∶10,000, Li-COR) and scanned using an Odyssey Infrared Imaging System (Li-COR). Relative intensities of protein bands normalized to actin were determined using Odyssey software (version 2.1).

### Statistical analyses

Values reported were expressed as means ± SEM. For two-group comparison (MRI analysis), two-sample *t*-test was employed. To determine interaction effects of iron deficiency and olfactory Manganese exposure as well as individual main effects, a two-way ANOVA was performed using Systat 13 (Systat). Differences were considered significant at *P*<0.05.
